# Navigating Cultural Stress and Mental Health: A Longitudinal Study of Parent‐Adolescent Dynamics Among Former Soviet Union Families

**DOI:** 10.1002/jad.12522

**Published:** 2025-05-23

**Authors:** Aigerim Alpysbekova, Seo Woo Lee, Carolina Scaramutti, Elena Bochkina, Tae Kyoung Lee, Cory L. Cobb, Pablo Montero‐Zamora, Duyen H. Vo, Sumeyra Sahbaz, Beyhan Ertanir, Lawrence Watkins, Evelyn O. Gualdron, Maya Benish‐Weisman, Hanit Ohana, Einat Elizarov, Seth J. Schwartz

**Affiliations:** ^1^ Department of Kinesiology & Health Education, College of Education University of Texas at Austin Austin Texas USA; ^2^ Department of Psychiatry and Behavioral Sciences, Miller School of Medicine University of Miami Coral Gables Florida USA; ^3^ Department of Psychology, Institute of Pedagogy and Psychology of Education Moscow City University (MCU) Moscow Russia; ^4^ Child Psychology and Education Sungkyungkwan University, South Korea Seoul South Korea; ^5^ Department of Health Behavior Texas A&M University College Station Texas USA; ^6^ Educational Psychology Universität Basel Basel Switzerland; ^7^ School of Social Work and Social Welfare The Hebrew University of Jerusalem Jerusalem Israel; ^8^ The Department of Counseling and Human Development The University of Haifa Haifa Israel

**Keywords:** cultural stress, Former Soviet Union, mental health, parent‐adolescent communication

## Abstract

**Introduction:**

Cultural stressors during migration can shape family dynamics and impact mental health outcomes. This study investigates the relationship between cultural stress, parent‐adolescent communication, and psychological well‐being among Former Soviet Union (FSU) immigrant families in Israel.

**Methods:**

This longitudinal survey study collected data at three time points. The first wave of data collection occurred between June and August 2020, recruiting families across Israel through social media, word‐of‐mouth, and referrals. Analyses were conducted on a final sample of 160 FSU immigrant adolescents (aged 12–15) and their parents, after accounting for attrition across waves. We used maximum likelihood estimation so that cases with missing data could be retained in analysis. Surveys assessed cultural stressors, parent‐adolescent communication, and mental health indicators (self‐esteem, hope, anxiety, and depressive symptoms).

**Results:**

Cultural distance was negatively associated with parental depressive symptoms but did not result in expected negative effects on adolescents. Parent‐adolescent communication at Time 2 mediated the relationship between cultural stress and adolescent well‐being. Additionally, discrimination experiences at Time 1 were positively associated with adolescents’ hope at Time 3.

**Conclusions:**

Despite the challenges posed by cultural stress, parent‐adolescent communication emerged as a protective factor, and was associated with lower levels of mental health issues. These findings highlight the importance of familial communication to support the well‐being of immigrant adolescents during acculturation.

## 1 Introduction

More than one million immigrants from the Former Soviet Union (FSU) have settled in Israel since the early 1990s after the collapse of the Soviet Union, accounting for approximately 15% of the country's total population. It is the second largest national diaspora in the country (Tartakovsky 2023). This group represents the largest single country‐of‐origin cohort within Israel's Jewish population (Borschel‐Dan 2016). Immigrants from the FSU, predominantly Jewish individuals from Russia, Ukraine, and other former Soviet republics, have introduced a diverse array of cultural, linguistic, and professional skills, enriching Israeli society (Al‐Haj 2002; Kostareva et al. 2020). However, the Israeli attitude towards these immigrants, particularly in the early years, was ambivalent (Remennick 2011).

Immigrants from the FSU were regarded as a valuable human resource because of their education and skills. Leshem and Lissak (2001) emphasized that between 1990 and 2000, the policy of multiculturalism partially displaced the assimilationist policy of immigrant absorption in Israel by attracting the new intellectual capabilities of people from the FSU who also desired to fit into the new society. This shift led to a gradual cultural blending with native Israelis, yet these immigrants were often perceived as having distinct cultural characteristics and difficulties adapting to Israeli social norms (Arellano‐Bover and San 2024).

In Israel, the phenomenon of migration is seen primarily as a return of Jews to their ancestral homeland rather than a move to a new land (Shuval 1998). FSU immigrant families in Israel often encounter cultural stressors, including discrimination and a negative context of reception—characterized by perceived hostility. This is because the local population often sees them not only as outsiders and a threat to their economic well‐being, but also as a threat to cultural values and national homogeneity (Ben‐Nun Bloom et al. 2015). Characteristics of migrants, including their ethnicity, motives for migration, religion and level of religiosity, were identified as significant factors contributing to anti‐immigrant sentiment (Semyonov et al. 2023). It is important to note that many migrants from the Former Soviet Union (FSU) were granted Israeli citizenship despite not being recognized as Jewish according to Halakhic (religious) definitions. This is reflected in the emergence of the “other” category under religion in Israeli national statistics, which includes tens of thousands of individuals—many likely non‐Jewish spouses or family members of Jewish immigrants—who entered under the Law of Return but are not classified as Jewish by religious authorities. (Remennick 2003). Moreover, the duality of Israel's state system must be considered. This is because it is based on both ethnicity and Western‐style democratic principles (Shafir and Peled 2002). The mismatch between these sometimes‐incompatible principles is particularly pronounced in the area of migration, having a significant impact on the process of integration of new citizens into society.

The aforementioned cultural stresses experienced by immigrants have a significant impact on their mental health (Meca and Schwartz 2024). As Tartakovsky and Schwartz (2001) and Tartakovsky and Baltiansky (2023). have demonstrated, immigrants from the former Soviet Union often arrive with expectations that their new society will accommodate their values and offer upward mobility. When these expectations are unmet, it can result in diminished group identification, economic difficulties, and a pervasive sense of isolation (Tartakovsky and Schwartz 2001; Tartakovsky and Baltiansky 2023). These challenges may contribute to heightened levels of anxiety, depression, and family tensions, particularly among adolescents navigating their dual identities within a new cultural environment (Gayman and Barragan 2013; Schwartz et al. 2014; Elizarov et al. 2023).

This study draws on cultural stress theory (Salas‐Wright and Schwartz 2019), which posits that immigrants’ psychological distress results from exposure to cultural stressors. According to cultural stress theory, immigrants face individual‐level stressors such as discrimination and negative context of reception (NCR), which likely predict problems in family functioning, such as communication breakdowns and conflicts (Schwartz et al. 2015). Such breakdowns in family functioning, in turn, predicts poor mental health outcomes such as low self‐esteem, increased anxiety, and depression among adolescents, as well as substance use and poor emotional regulation (Lorenzo‐Blanco et al. 2016; Qiao et al. 2024; Wang et al. 2025; Zhang et al. 2023). Prior studies with U.S. Latino populations support these pathways (Lorenzo‐Blanco et al. 2016; McCord et al. 2018), but few have tested them in other immigrant contexts.

Indeed, family functioning plays a key mediating role in cultural stress theory (Meca and Schwartz 2024). Cultural stressors may disrupt family communication and cohesion, worsening adolescent outcomes. Although some studies (e.g., García et al. 2024; Lorenzo‐Blanco et al. 2016) have explored these effects in other migrant groups, few have examined parent‐adolescent interactions or included parent outcomes in these models.

Further, findings testing cultural stress theory have typically been limited to adolescents’ reports, with few studies examining the full scope of parent‐adolescent interactions or measuring parental outcomes. For instance, studies have and found that cultural stressors linked to migration, such as those experienced after Hurricane Maria, predicted family conflict (García et al. 2024) and impaired mental health (Montero‐Zamora et al. 2023) in Puerto Rican families. However, their studies were limited in their ability to fully explore complex mediation effects of cultural stress on parent and adolescent outcomes through family functioning. Although FSU immigrants in Israel and Latino immigrants in the United States come from distinct cultural and historical backgrounds, they share key migration‐related challenges, such as navigating multiple cultural streams, adapting to new societal norms, and facing discrimination.

Cultural stress theory has mainly been studied in Hispanic populations in the United States, but the Israeli context offers a unique case. Unlike Latino migrants in the U.S., who often face stigma, FSU immigrants in Israel share a Jewish identity with the majority population (Heilbrunn et al. 2016). This shared identity may reduce certain cultural stressors, such as language barriers or religious discrimination, allowing for a different understanding of migration‐related challenges. A number of studies have identified a correlation between the linguistic environment and the level of stress experienced by migrants. Research findings have indicated that migrants from the FSU who possess proficiency in English experience reduced levels of stress in comparison to those who speak Russian as their primary means of communication (Kayam and Hirsch 2014). However, they still face significant cultural distance in terms of their upbringing in the Soviet Union and the sociocultural norms of Israeli society, which may lead to social exclusion or marginalization. For instance, although Jewish Israelis may be more accepting of FSU immigrants due to shared religion, there are tensions, especially given the large influx of FSU Jews in Israel over the past few decades (Al‐Haj 2002; Pew Research Center 2016).

Although some segments of the Israeli population are welcoming, many FSU immigrants still face discrimination that can affect their mental health, family functioning, and integration into Israeli society (Elizarov et al. 2023; Titzmann et al. 2011; Yakhnich et al. 2025). The perceived distance from Israeli culture, whether due to language differences, unfamiliarity with Israeli social customs, or experiences of exclusion, may contribute to increased stress among FSU immigrant parents. Understanding these dynamics is crucial, as family functioning plays a central role and is associated with lower levels of migration‐related stress (Cox and Paley 1997).

## 2 The Present Study

This study extends cultural stress theory by testing its applicability to the Former Soviet Union (FSU) population in Israel, focusing on longitudinal associations over three waves. We hypothesize following:

Hypothesis 1a Higher adolescent reports of cultural stress will predict lower subsequent adolescent reports of parent‐adolescent communication.

Hypothesis 1b Higher parent reports of cultural stress will predict lower subsequent parent reports of parent‐adolescent communication.

Hypothesis 2a Higher adolescent reports of parent‐adolescent communication will predict better subsequent adolescent and parent mental health and well‐being.

Hypothesis 2b Higher parent reports of parent‐adolescent communication will predict better subsequent adolescent and parent mental health and well‐being.

Hypothesis 3a Adolescent reports of parent‐adolescent communication will mediate the relationship between adolescent‐reported cultural stress and adolescent and parent mental health and well‐being.

Hypothesis 3b Parent reports of parent‐adolescent communication will mediate the relationship between parent‐reported cultural stress and adolescent and parent mental health and well‐being.

To our knowledge, the present study is among the first studies to examine cultural stress theory outside the United States, incorporating both adolescent and parent perspectives on cultural stressors, communication, mental health and well‐being.

## 3 Methods

### 3.1 Study Design and Participants

Data was collected in three timepoints. The first wave of data collection occurred between June and August 2020, families across Israel were recruited for the study through social media, word‐of‐mouth, and referrals. Data collection for Time 1 took place from June to December 2020, with two subsequent assessments occurring every 6 months. Eligibility criteria required that (a) each family include one parent and one adolescent aged 12–15 who were willing to participate, (b) family have immigrated to Israel from a former Soviet country (e.g., Russia, Ukraine) within the 5 years preceding the assessment, and (c) both parent and adolescent were proficient in Russian or Hebrew. Eligibility was confirmed during a phone call with a member of the research team, after which families received the study questionnaires. As a token of appreciation, parents received $23, and adolescents received $10 for their participation. Ethical guidelines ensure fair compensation for minors, while parents receive higher compensation for facilitating participation and providing consent. The study's longitudinal design included follow‐ups at 6‐month intervals, tracking changes in FSU immigrant families’ experiences over time. Families completed self‐report surveys online independently, with reminders and incentives used to reduce attrition. The final sample consisted of 160 FSU ‐parent‐adolescent dyads in Israel. Demographic details are provided in Table 1. The study was approved by the ethics committee at the Hebrew University of Jerusalem (approval number 133/20).

**Table 1:** Participants demographic information (*N* = 160).

Variable	M (SD) or %	Minimum/Maximum
Parents		
– Female	87.50%	
– Age	41.75 (5.25)	30/50
Relationship with child (not respond: 3.1%)		
– Mother	85.6%	
– Father	10.6%	
– Stepfather	0.6%	
Educational Level		
– High School (including not completed)	61.0%	
– College (including graduate college)	39.0%	
Marital status		
– Married	76.3%	
– Divorced/living separately/never married	21.4%	
Adolescents		
– Females	49.4%	
– Age	13.61 (1.25)	11/17
– Median grade	8th grade	6th grade/11th grade
Household information		
Country of origin		
– Russia	60.6%	
– Ukraine	34.4%	
– Belarus	4.4%	
Years of immigration to Israel	2.63 (1.48)	0.00/5.00
Family (monthly) income (unit: Shekel [equivalent to $.30]; not respond: 2.5%)		
– Less than 10,000	48.1%	
– 10,000–15,000	37.5%	
– 15,000–20,000	10.0%	
– Over 20,000	1.9%	
Numbers of children in house (Median)	2	1/4
People to room ratio^a^	1.46	

^a^ The number of people living in the home is divided by the number of bedrooms in the home. ^b^Any household members. ^c^June 16, 2020 to August 17, 2020.

### 3.2 Preliminary Analysis

Table 1 provides an overview of participant demographic characteristics. The majority of families (60.6%) originated from Russia, followed by Ukraine (34.4%) and Belarus (4.4%). The average number of years since immigration to Israel was 2.63 years (SD = 1.48), with a range from 0 to 5 years. Specifically, 87.5% (*N* = 140) of parents were female, and their average age was 41.75 years (SD = 5.25), with ages ranging from 30 to 50 years. In terms of education, 61.0% of parents had completed high school (including those who did not graduate), and 39.0% had attended or completed college. Regarding marital status, 76.3% of parents were married, while 21.4% were divorced or living separately. Adolescents (females = 49.4%) in the study were 13.61 years (SD = 1.25) on average, with ages ranging from 11 to 17 years. The median year in school for adolescents was 8th grade, with grades ranging from 6th to 11th grade.

### 3.3 Measures

All the scales used in the present study were translated and back translated from English into Russian or Hebrew and administered in the participant's preferred language. We computed all subscale scores as the sum of the corresponding item responses. Reliability estimates are reported for the wave in which each variable is used in our analytic model.

### 3.4 Cultural Stress

We assessed cultural stress in terms of discrimination, negative context of reception, and perceived cultural distance.

#### 3.4.1 Discrimination

Discrimination was measured using a 7‐item scale (Phinney et al. 1998; α = 0.92 for parents and α = 0.89 for adolescents) that assesses perceived discrimination in different contexts. Sample items include “I feel that people treat me unfairly because of my background.” Responses range from 1 (*Strongly Disagree*) to 5 (*Strongly Agree*).

#### 3.4.2 Negative Context of Reception

We used the 6‐item Negative Context of Reception Scale (Schwartz et al. 2014) to evaluate individuals’ perceptions of the opportunities and welcomeness they were provided in Israel society. This scale utilizes a 5‐point Likert scale ranging from 0 (*Strongly Disagree*) to 4 (*Strongly Agree*). Items were adapted to reflect the Israeli context. Sample Items include “I don't have the same chances in life here as people from other countries.” Cronbach's alpha was 0.87 for parents and 0.85 for adolescents.

#### 3.4.3 Cultural Distance

Cultural distance was assessed using a 7‐item scale (Demes and Geeraert 2013; α = 0.86 for parents and α = 0.82 for adolescents) measuring the perceived cultural gap between migrants and the host community. The scale includes items such as “I find it difficult to relate to the culture here.” Responses range from 1 (*Very Difficult*) to 7 (*Very Easy*).

#### 3.4.4 Parent–Adolescent Communication

Parent‐adolescent communication served as our indicator of family functioning. It was measured using the Parent‐Adolescent Communication Scale (Barnes and Olson 1982; 10 items, α = 0.88 for parents and α = 0.93 for adolescents). A sample item is “How often do you talk to your child/parent about personal feelings?” The response scale ranged from 1 (*Strongly Disagree*) to 5 (*Strongly Agree*).

### 3.5 Mental Health and Well‐Being

Mental health and well‐being among parents and adolescents were assessed in terms of self‐esteem, optimism, anxiety, and depressive symptoms.

#### 3.5.1 Self‐Esteem

We assessed self‐esteem using the Rosenberg (1965) Self‐Esteem Scale (10 items, α = 0.86 for parents and α = 0.92 for adolescents). An example item is “I take a positive attitude toward myself.” Responses were on a 4‐point Likert scale from 1 (*Strongly Disagree*) to 4 (*Strongly Agree*).

#### 3.5.2 Optimism

**Optimism** for adolescents was assessed using the Children's Hope Scale (Snyder et al. 1997; six items, α = 0.86). A sample item is “I think I am doing well in life.” Response choices ranged from 1 (*None of the Time*) to 6 (*All of the Time*). For parents, optimism was assessed with the Life Orientation Test–Revised (Scheier and Carver 1985; 10 items, α = 0.79), with items such as “I'm always optimistic about my future.” Responses ranged from 1 (*Strongly Disagree*) to 5 (*Strongly Agree*).

#### 3.5.3 Anxiety

**We** assessed anxiety using the Generalized Anxiety Disorder Scale −7 (Spitzer et al. 2006; seven items, α = 0.86 for parents and α = 0.90 for adolescents). A sample item is, “How often do you feel worried too much about different things?” The response scale ranged from 0 (*Not at All*) to 3 (*Nearly Every Day*).

#### 3.5.4 Depressive Symptoms

**Depressive symptoms** for both parents and adolescents were assessed using the Centers for Epidemiologic Studies–Depression Scale (Grzywacz et al. 2006; 10 items, α = 0.88 for parents and α = 0.85 for adolescents). A sample item is “I felt lonely.” Responses ranged from 0 (*Rarely or None of the Time*) to 3 (*All of the Time*).

### 3.6 Covariates

Adolescent age (continuous), adolescent sex (categorical), parent sex (categorical), time of arrival in Israel (ordinal), the country of origins (categorical, with Russian as the reference group), and prior levels of parent‐adolescent communication and mental health and well‐being among parents and adolescents were controlled.

### 3.7 Data Analysis

Descriptive statistics and bivariate correlations were computed as the first step of the analysis. Second, to investigate the relationships among cultural stress, parent‐adolescent communication, and mental health and well‐being among parents and adolescents, the current study employed a longitudinal structural equation modeling (SEM; Little 2024) with three waves of data collection in a sample of 160 parent‐adolescent dyads. SEM allows for the simultaneous examination of complex associations among multiple variables, including both observed and latent constructs (Little 2024). We entered the three cultural stressors as observed variables, because perceived cultural distance was not strongly correlated with discrimination and with negative context of reception.

Given the small sample size (*N* = 160), we estimated separate SEM models for adolescent‐reported and parent‐reported cultural stressors and family communication. Parent and adolescent reported outcomes were included in both models. In the first model (referred to as the adolescent communication model), *adolescent‐reported* parent‐adolescent communication was allowed to mediate the predictive effects of adolescents’ cultural stressors on both adolescents’ and parents’ well‐being and mental health. In the second model (referred to as parent communication model), *parent‐reported* parent‐adolescent communication was allowed to mediate the predictive effects of *parents’* cultural stressors on both adolescents’ and parents’ well‐being and mental health. All possible indirect effects from cultural stress to mental health and well‐being among parents and adolescent were estimated within each model. Standardized coefficients (βs) are reported as effect sizes (Cohen 1988). The average rate of missing data across Waves 2 through 6 was 27.04% (ranging from 10.4% at Wave 1 to 43.1% at Wave 3). Little (1988) missing completely at random (MCAR) test was performed, and results indicated significant chi‐square value, $\chi^{2}$(295) = 399.10, *p* < 0.001. However, the MCAR test is known to be sensitive, particularly when sample sizes are small or there is substantial missing data, as even minor deviations from randomness can result in a finding of “not random” (Little 1988). The normed chi‐square value (χ^2^/df) accounts for this sensitivity, and if the normed value is below 2, the MCAR assumption can be retained (Alavi et al. 2020). The normed chi‐square value was 1.3, suggesting that the MCAR assumption can be retained.

Full Information Maximum Likelihood (FIML) was employed, and the robust maximum likelihood (MLR) estimator was used, as a way of handling non‐normality and missing data. The goodness of fit SEM models was evaluated using the Comparative Fit Index (CFI), Tucker‐Lewis index (TLI), Root Mean Square Error of Approximation (RMSEA), and Standardized Root Mean Square Residual (SRMR). CFI/TLI values of 0.90 or above, and RMSEA and SRMR values of 0.08 or below, suggest an acceptable fit (Little 2024). The chi‐square statistic is reported, though it is not used for model interpretation. The analyses were conducted using SPSS version 29 and M*plus* version 8.5 (Muthén and Muthén 2017).

## 4 Results

Correlations among study variables are presented in Table 2. Among the cultural stressors at Time 1, adolescent‐reported negative context of reception at Time 1 was negatively correlated with adolescents’ own reports of parent‐adolescent communication at Time 2. However, parent‐reported cultural stressors were not significantly correlated with parents’ own reports of parent‐adolescent communication. Adolescent report of discrimination at Time 1 was positively correlated with adolescent anxiety and negatively correlated with adolescent and parent optimism at Time 3. Parent report of discrimination at Time 1 was positively correlated with parent depressive symptoms and parent anxiety and also positively correlated with adolescent optimism at Time 3. Adolescent‐reported parent‐adolescent communication at Time 2 was correlated with adolescent reports of mental health and well‐being at Time 3. Parent‐reported parent‐adolescent communication at Time 2 was positively correlated with both parent and adolescent reports of self‐esteem at Time 3.

**Table 2:** Bivariate correlations among study variables (*N* = 160).

Variable	1	2	3	4	5	6	7	8	9	10	11	12	13	14	15	16
1. Cultural distance (Adol T1)	—															
2. Cultural distance (Parent T1)	0.24**	—														
3. Discrimination (Adol, T1)	−0.12	−0.20*	—													
4. Discrimination (Parent, T1)	−0.05	−0.00	0.05	—												
5. NCR (Adol, T1)	0.00	−0.04	0.47***	0.00	—											
6. NCR (Parent, T1)	−0.03	−0.12	0.02	0.48***	0.19	—										
7. PAC (Adol, T2)	0.01	0.00	−0.06	−0.06	−0.29***	−0.15	—									
8. PAC (Parent, T2)	0.10	0.16	0.15	−0.15	−0.15	−0.08	0.46***	—								
9. Depression (Adol, T3)	−0.07	−0.10	0.09	−0.12	0.16*	0.00	−0.31***	−0.15	—							
10. Depression (Parent, T3)	0.06	0.00	0.10	0.33***	0.05	0.31***	0.04	−0.04	−0.06	—						
11. Anxiety (Adol, T3)	−0.16	−0.12	0.44***	−0.05	0.32***	0.05	−0.26**	−0.10	0.77***	0.01	—					
12. Anxiety (Parent, T3)	0.02	0.04	0.08	0.24**	0.08	0.17	0.04	−0.06	−0.18*	0.70***	−0.07	—				
13. Optimism (Adol, T3)	0.10	−0.13	−0.20*	0.22*	−0.31***	−0.03	0.41***	0.08	−0.52***	0.04	−0.42***	−0.10	—			
14. Optimism (Parent, T3)	0.20*	−0.01	−0.19*	−0.09	−0.10	0.09	0.06	0.09	−0.10	0.06	−0.19	0.08	0.14	—		
15. Self‐esteem (Adol, T3)	0.05	−0.17	0.11	0.11	−0.24**	−0.04	0.45***	0.25**	−0.47***	0.03	−0.38***	−0.11	0.77***	0.10	—	
16. Self‐esteem (Parent, T3)	−0.16	−0.10	−0.14	−0.14	−0.17	−0.07	0.08	0.25**	−0.03	−0.24**	−0.12	−0.26**	0.10	−0.07	0.30**	—
Mean	24.35	20.91	12.76	12.30	12.77	14.39	37.25	38.94	13.89	18.59	12.07	11.35	21.84	18.43	19.77	20.84
SD	7.99	7.96	5.86	5.80	14.39	4.32	8.39	5.44	10.11	5.48	5.37	3.87	4.86	1.77	3.93	2.65
Missing N (%)	25 (11.8)	33 (15.6)	22 (10.4)	30 (14.2)	25 (11.8)	31 (14.7)	66 (31.3)	58 (27.5)	26 (12.3)	82 (38.9)	91 (43.1)	81 (38.4)	90 (42.7)	82 (38.9)	90 (42.7)	81 (38.4)
Skewness	0.51	0.73	1.67	1.76	0.31	0.14	−0.69	−0.20	0.12	0.91	1.12	1.31	−0.72	−0.62	−0.95	−0.26
Kurtosis	−0.20	0.56	2.61	4.72	−0.03	−0.21	1.14	0.30	−1.13	1.02	0.40	2.50	1.59	0.74	1.40	1.05

Abbreviations: Adol, adolescent; T, Time; NCR, negative context of reception; PAC, parent‐adolescent communication.

* *p* < 0.05

** *p* < 0.01

*** *p* < 0.001.

Among the 120 bivariate correlations, 35 (30%) were statistically significant at *p* < 0.05, *p* < 0.01, or *p* < 0.001. Most of these significant associations were small to moderate in magnitude (ranging from *r* = 0.16 to *r* = 0.47), particularly among cultural stress variables at Time 1 and well‐being indicators at Time 3. For example, adolescent‐reported discrimination was positively correlated with adolescent anxiety (*r* = 0.44, *p* < 0.001) and negatively correlated with optimism (*r* = –0.20, *p* < 0.05). Parent‐reported discrimination was positively correlated with parent depressive symptoms (*r* = 0.33, *p* < 0.001) and parent anxiety (*r* = 0.24, *p* < 0.01). These patterns suggest modest but meaningful associations between cultural stress and mental health, underscoring the value of examining mediated pathways through parent‐adolescent communication.

### 4.1 Adolescent Communication Model

**Figure 1:**
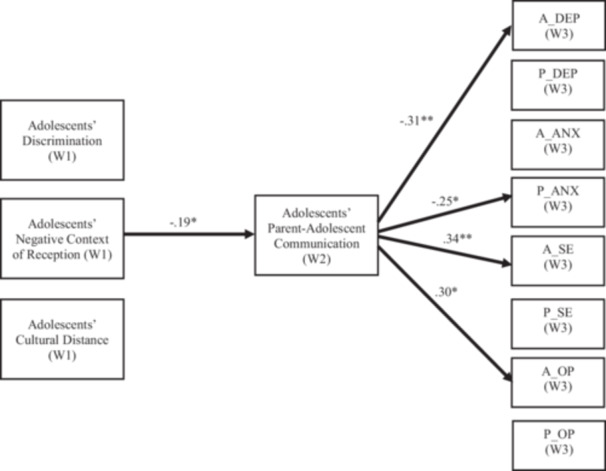
Adolescent communication model. *Note:* Only statistically significant paths are shown. A = adolescents, ANX = anxiety, DEP = depressive symptoms, OP = optimism, P = parents, SE = self‐esteem, W = wave. *p < 0.05; **p < 0.01.

The model with the adolescent report of parent‐adolescent communication as a mediator was shown in Figure 1. The model demonstrated an acceptable fit to the data, χ²(148) = 190.58, *p* = 0.01; CFI = 0.93; TLI = 0.90; RMSEA [90% CI] = 0.05 [0.02, 0.06]; SRMR = 0.08. Given the large number of paths, Table 3 presents only significant standardized path estimates. Results indicated that only adolescent reports of negative context of reception at Time 1 negatively predicted adolescent‐reported parent‐adolescent communication at Time 2 (β = −0.19, *p* < 0.05, 95% CI [ − 0.38, −0.01]). Adolescent reports of cultural stressors at Time 1 did not directly predict their mental health and well‐being at Time 3. Adolescent reports of parent‐adolescent communication at Time 2 negatively predicted their own depressive symptoms (β = −0.31, *p* < 0.01, 95% CI [ − 0.49, −0.12]) and anxiety (β = −0.25, *p* < 0.05, 95% CI [ − 0.45, −0.05]) at Time 3, and positively predicted their self‐esteem (β = 0.34, *p* < 0.01, 95% CI [0.09, 0.60]) and hope (β = 0.30, *p* < 0.05, 95% CI [0.04, 0.57]) at Time 3. None of the hypothesized mediated effects of adolescent‐reported cultural stressors on adolescent and parent outcomes through adolescent‐reported parent‐adolescent communication were significant. In terms of covariates, adolescent age is positively associated with adolescent‐reported parent‐adolescent communication at Time 2, β = 0.13, *p* < 0.05, 95% CI [0.03, 0.22]. Adolescents from countries other than Russia, Belarus, or Ukraine reported higher levels of depressive symptoms compared to those from Russia, β = 0.15, *p* < 0.03, 95% CI [0.001, 0.21].

**Table 3:** Standardized path estimates for significant paths in the adolescent communication model (*N* = 160).

Path	(Estimate β)	SE
**Adolescent Cultural Stressors to Adolescent Communication (T1‐T2)**		
Adolescent Negative Context of Reception T1→ Adolescent Communication T2	−0.19*	0.09
**Adolescent Communication to Outcomes (T2‐T3)**		
Adolescent Communication T2→ Adolescent Depression T3	0.12*	0.06
Adolescent Communication T2→ Adolescent Anxiety T3	−0.25*	0.10
Adolescent Communication T2→ Adolescent Self‐Esteem T3	0.34**	0.13
Adolescent Communication T2→ Adolescent Optimism T3***	0.30*	0.14

* *p* < 0.05

** *p* < 0.01

*** *p* < 0.001.

### 4.2 Parent Communication Model

**Figure 2:**
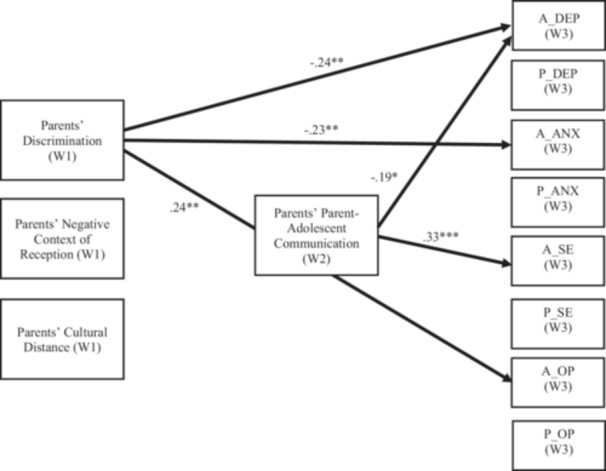
Parent communication model. *Note:* Only statistically significant paths are shown. A = adolescents, ANX = anxiety, DEP = depressive symptoms, OP = optimism, P = parents, SE = self‐esteem, W = wave. *p < 0.05; **p < 0.01; ***p < 0.001.

Results for the model with parent reports of parent‐adolescent communication as a mediator are shown in Figure 2. The model provided an acceptable fit to the data, χ²(139) = 176.88, *p* = 0.02; CFI = 0.94; TLI = 0.92; RMSEA [90% CI] = 0.04 [0.02, 0.06]; SRMR = 0.07. Table 4 presents significant standardized path estimates among parent‐reported of cultural stressors, parent‐reported parent‐adolescent communication, and parent and youth reports of mental health and well‐being. The results indicated that parents’ cultural stressors at Time 1 did not predict parent‐reported parent‐adolescent communication at Time 2. Parent reports of discrimination at Time 1 directly and negatively predicted adolescent depressive symptoms (β = −0.24 *p* < 0.01, 95% CI [−0.40, −0.07]) and adolescent anxiety (β = −0.23, *p* < 0.01, 95% CI [−0.39, −0.07]), and positively predicted adolescent hope (β = 0.24, *p* < 0.01, 95% CI [0.06, 0.43]) at Time 3. Parent‐reported parent‐adolescent communication at Time 2 negatively predicted adolescent depressive symptoms (β = −0.19, *p* < 0.05, 95% CI [−0.37, −0.11]) and positively predicted adolescent self‐esteem (β = 0.33, *p* < 0.001, 95% CI [0.18, 0.48]) at Time 3. No significant predictive mediated effects of parent reports of cultural stressors on parent and adolescent outcomes through parent‐reported parent‐adolescent communication emerged. Regarding covariate effects, parents from Ukraine, and from countries other than Russia, Ukraine, or Belarus, reported higher levels of parent‐adolescent communication compared to those from Russia (β = 0.14, *p* < 0.05, 95% CI [0.04, 0.23]).

**Table 4:** Standardized path estimates for significant paths in the parent communication model (*N* = 160).

Path	(Estimate β)	SE
**Parent Cultural Stressors to Outcomes (T1‐T3)**		
Parent Discrimination T1→ Adolescent Depression T3	−0.24**	0.09
Parent Discrimination T1→ Adolescent Anxiety T3	−0.23**	0.08
Parent Discrimination T1→ Adolescents Optimism T3		
**Parent Communication to Outcomes (T2‐T3)**		
Parent Communication T2→ Adolescent Depression T3	−0.19*	0.09
Parent Communication T2→ Adolescent Self‐Esteem T3	0.33***	0.08

* *p* < 0.05

** *p* < 0.01

*** *p* < 0.001.

## 5 Discussion

The present study examines the associations among cultural stressors, parent‐adolescent communication, and mental health outcomes among FSU families living in Israel. Our findings suggest several patterns that are consistent with theoretical expectations (e.g., cultural stress predicts mental health outcomes), but also some unexpected results that warrant further exploration (e.g., no mediating effect of family communication in the associations of cultural stress with mental health and well‐being outcomes). It is also noteworthy that we used perceived cultural distance, which has been defined as a cultural stressor (Demes and Geeraert 2013) but had not previously been studied alongside discrimination and negative context of reception.

In terms of the mediating role of Time 2 adolescent communication, we found several important insights regarding the predictive effects of Time 1 cultural stressors on Time 3 mental health and well‐being. Specifically, although the mediational paths were not statistically significant, parent‐adolescent communication was significantly predicted by negative context of reception (for adolescents) and by discrimination (for parents)—and parent‐adolescent communication from both reporters predicted adolescent self‐esteem (positively) and depressive symptoms (negatively). Adolescent‐reported communication also significantly predicted parent optimism (positively) and anxiety (negatively). As a result, the majority of the predictive effects of parent‐adolescent communication operated across reporters—adolescent‐reported communication predicting parent‐reported outcomes, and vice versa. This pattern has not been reported in other samples. However, these findings do support previous research suggesting that open, supportive family communication can promote well‐being among family members (Lessard et al. 2023).

Additionally, Time 1 parent‐reported discrimination predicted Time 3 adolescent‐reported depressive symptoms, anxiety, and hope—but in an opposite direction than hypothesized. Specifically, adolescents whose parents reported greater levels of discrimination were more likely to report being more hopeful, less anxious, and less depressed. Although these findings appear counterintuitive, they may suggest a form of family resilience, where adolescents watch their parents experience and handle discrimination and are inspired by how the parents are able to manage these experiences. Indeed, in their work on family resilience among Hispanic immigrant adolescents in the United States, Smokowski and Bacallao (2011) found that adolescents were often proud of their parents for withstanding culturally stressful experiences and conditions—and that youth derived strength from their parents’ resilience. Additional mixed‐method research is needed to determine whether a similar explanation may apply to the present sample.

We found parent‐reported communication at Time 2 positively predicted adolescents’ self‐esteem, and negatively predicted depressive‐symptoms, at Time 3. We also found that adolescents’ reports of communication at Time 2 predicted adolescents’ own reports of self‐esteem, optimism, and depressive symptoms, as well as parent reports of anxiety, in the expected directions. These findings are consistent with family systems theory (Cox and Paley 1997). According to this theory, families function as dynamic systems, where effective communication enhances emotional support, cohesion, and psychological well‐being across members (Minuchin 2009). Open and supportive communication between parents and adolescents is associated with lower level of acculturative stress and reinforcing family solidarity during the migration process. Moreover, effective parent communication may help parents themselves feel more connected and engaged in their children's well‐being, which can reinforce their own sense of optimism and purpose. Parent‐adolescent communication is thus crucial in protecting against the external stresses of migration and acculturation, and in serving as an important resource for both adolescents’ mental health and parents’ psychological well‐being (Hoskins et al. 2023).

In terms of covariate effects, a significant finding in the *adolescent communication model* is the positive relationship between adolescent age and their perceptions of parent‐youth communication at Time 2. Specifically, older adolescents (i.e., those further in high school) reported more effective communication with their parents. This result is consistent with previous research suggesting that as adolescents age, they develop better communication skills due to the maturation of cognitive and emotional regulation abilities (Pöpplau et al. 2023). Early adolescence may also be a more difficult time for parent‐adolescent relationships (Chiang et al. 2023).

It is important to note that the literature on FSU immigrant families in Israel remains relatively limited. Most research on cultural stress, parent‐adolescent communication, and adjustment has been conducted with other populations and in different settings. Indeed, the majority of psychologically oriented research, including studies on adolescents and families, originates from countries such as the United States, Canada, Australia, and Western Europe. As a result, it is unclear whether some of the unexpected findings in this study represent the “rule” or the “exception”– would these results be replicated in other groups and contexts? Alternatively, it is possible that different patterns may be observed with different migrant groups and destination contexts.

Clearly, further research is needed to explore cultural stress and family dynamics among FSU immigrant families in Israel, particularly in light of the Russian invasion of Ukraine (which occurred after the present cohort was recruited) and the subsequent migration of millions of Ukrainian families worldwide, including to Israel. A key avenue for future research involves cross‐cultural comparative studies examining FSU immigrant families in Israel and in other countries. Although Titzmann et al. (2011) have contributed valuable work in this area, much more remains to be done.

### 5.1 Limitations and Future Directions

The present findings should be interpreted considering at least four important limitations. First, the sample size was relatively small, and most participating parents were mothers. These sample size issues did not permit us to systematically examine the moderating effects of potential moderating variables, including parent gender (fathers vs. mothers) in our findings. Second, the study covered a relatively short time span of approximately 1 year, leaving open the question of whether different patterns might emerge over longer periods. Third, the present study was conducted during the COVID‐19 pandemic, a time when most families were under quarantine and experiencing atypical social dynamics. It remains unclear whether the findings would differ under more typical social conditions. Fourth, all data were gathered through self‐reports, which may introduce biases related to subjective perceptions or reporting tendencies. Although the use of both parent and youth reports helps to overcome biases attributable to shared method variance where all variables are reported by the same person (Podsakoff et al. 2024), including some objective or observational measures might have been helpful. We also cannot draw causal conclusions because our design was not experimental.

An additional limitation involves our inability to utilize advanced family‐based analytic strategies, such as actor‐partner interdependence modeling (Kenny 2018), because of our somewhat small sample size. Indeed, the actor‐partner approach requires that both youth and parent reports of all predictors be allowed to predict both parent and adolescent reports of the mediating mechanism (parent‐adolescent communication), and that both parent and adolescent reports of parent‐adolescent communication be allowed to predict all of the outcome variables. Additional paths are required for covariates, such as adolescent and parent sex, age, years lived in Israel, and country of birth. Estimating more paths than one's sample size can accommodate can lead to unstable path estimates (Kline 2023). Future work should endeavor to recruit and follow larger samples so that more elegant analytic approaches can be utilized.

In summary, despite these and other limitations, the present study examined the effects of cultural stress, parent‐adolescent communication, and mental health and well‐being among FSU parents and adolescents in Israel. While cultural stressors generally appeared to undermine family communication and adjustment, some findings suggest potential resilience mechanisms, wherein parents experiencing cultural stress may foster strength and resilience in their adolescent children. These patterns highlight unexpected effects of cultural stress and underscore the need for further investigation. We hope this study inspires additional research in this important area.

## Ethics Statement

The study was approved by the Ethics Committee at the Hebrew University of Jerusalem (approval number 133/20), ensuring compliance with ethical research guidelines.

## Consent

Informed consent was obtained from all participants and their legal guardians data collection.

## Conflicts of Interest

The authors declare no conflicts of interest.

## Data Availability

The data supporting the findings of this study are available upon reasonable request from the corresponding author.
